# The RDoC approach for translational psychiatry: Could a genetic disorder with psychiatric symptoms help fill the matrix? the example of Prader–Willi syndrome

**DOI:** 10.1038/s41398-020-00964-6

**Published:** 2020-08-08

**Authors:** Juliette Salles, Emmanuelle Lacassagne, Grégoire Benvegnu, Sophie Çabal Berthoumieu, Nicolas Franchitto, Maithé Tauber

**Affiliations:** 1 Université de Toulouse III, Toulouse, F-31000 France; 2 CHU Toulouse, Service de psychiatrie et psychologie, psychiatrie, Toulouse, F-31000 France; 3 Inserm Unité 1043, CNRS 5828, Université Paul Sabatier, Toulouse III, France; 4 Institut des Handicaps Neurologiques, Psychiatriques et Sensoriels- CHU de Toulouse, Toulouse, F-31000 France; 5 Centre de référence du Syndrome de Prader Willi et autres syndromes avec troubles du comportement alimentaire Unité d’endocrinologie, obésité, maladies osseuses, génétique et gynécologie médicale Hôpital des enfants CHU Toulouse, Toulouse, France; 6 CHU Toulouse, Service d’addictologie clinique, urgences réanimation médecine, Toulouse, F-31000 France

**Keywords:** Clinical genetics, Addiction

## Abstract

The Research Domain Criteria project (RDoc) proposes a new classification system based on information from several fields in order to encourage translational perspectives. Nevertheless, integrating genetic markers into this classification has remained difficult because of the lack of powerful associations between targeted genes and RDoC domains. We hypothesized that genetic diseases with psychiatric manifestations would be good models for RDoC gene investigations and would thereby extend the translational approach to involve targeted gene pathways. To explore this possibility, we reviewed the current knowledge on Prader–Willi syndrome, a genetic disorder caused by the absence of expression of some of the genes of the chromosome 15q11–13 region inherited from the father. Indeed, we found that the associations between genes of the PW locus and the modification identified in the relevant behavioral, physiological, and brain imaging studies followed the structure of the RDoC matrix and its six domains (positive valence, negative valence, social processing, cognitive systems, arousal/regulatory systems, and sensorimotor systems).

## Introduction

The current diagnostic systems in psychiatry, the International Classification of Diseases (ICD) and the Diagnostic and Statistical Manual of Mental Disorders (DSM), were developed to provide a common language to define psychiatric disorders. These systems improved psychiatric diagnosis by specifying symptom clusters and offering standardized categorization across the world. Nevertheless, this diagnostic approach is based only on symptoms and thus presents some limitations, especially for a pathophysiological approach. The inclusion of biomarkers was a request for the last edition of the DSM but this did not occur. Although the diagnostic categories have been built from clearly defined symptoms, they have continued to show considerable clinical heterogeneity, which has made it difficult to determine the significant biomarkers^1^. An alternative of the categorical approach is the dimensional approach. This approach was discussed deeply for the DSM-5, but it was finally not chosen. However it is considered particularly relevant for NeuroDevelopmental Disorder (NDD)^2,3^.

To address the need for a new approach to classifying mental disorders, the National Institutes of Mental Health (NIMH) launched the Research Domain Criteria project (RDoC). The ultimate goal of this project is prediction medicine for psychiatry, which means a diagnostic system based on a deeper understanding of the biological and psychosocial bases of a group of disorders^4^. The current version of the RDoC matrix is constructed around six major domains: negative valence systems, positive valence systems, cognitive systems, systems for social processes, arousal/regulatory systems, and sensorimotor systems. The RDoC matrix is used to investigate circuit-based functional dimensions across multiple units of analysis (circuits, physiology, behavior, and self-reports) both synchronically and diachronically, this last through the neurodevelopmental dimension. In addition, the two additional aspects of neurodevelopmental trajectories and interactions with the environment are equally important elements of the RDoC framework.

Despite the clear relevance of gene investigations that include neurodevelopment and environmental interactions (epigenetic), the references to specific genes had to be removed from the RDoC matrix indeed psychiatric disorders are complex disorders and genetic variance (major gene and common variation) do not explain transmission as a whole^5^. Genetic investigations have thus continued to be a major challenge for psychiatric research. For this reason, we hypothesized that genetic disorders presenting psychiatric symptoms would provide valuable information to expand the RDoC matrix, especially by connecting the RDoC domains to well-identified mutations. Moreover, some genetic disorders can be diagnosed just after birth, offering the possibility of neurodevelopmental investigation.

**Fig. 1:**
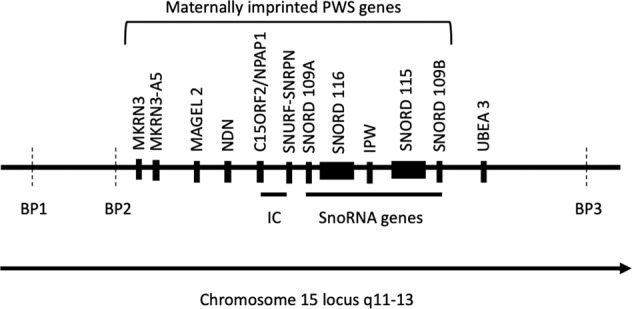
PWS region in chromosome 15. The PWS domain between the two common proximal breakpoints (BP 2 and BP 3) contains paternally expressed protein-coding genes (MKRN3, MKRN3-A5, MAGEL2, NDN, snoRNAs, IPW and SNRPN-SNURF, C15orf2) and the antisense transcript to UBE3A. *BP* Break Point; *IC* Imprinting Center.

Prader–Willi syndrome (PWS) is a rare genetic neurodevelopmental disorder resulting from a paternally inherited gene mutation, including the lack of expression of several imprinted genes of the chromosome 15 locus q11-q13^6^ region (Fig. 1). At birth, PWS patients present hypotonia with impaired feeding behavior comprising sucking and swallowing deficits and anorexia, which lead to failure to thrive. Conversely, in childhood and adulthood, the patients display hyperphagia and poor satiety, which leads to early severe obesity with endocrine dysfunction (impaired sexual development and growth, central hypothyroidism) and intellectual disabilities. This syndrome is also associated with psychiatric manifestations, including symptoms that can be connected with several DSM and ICD diagnoses, such as intellectual disability, autism spectrum disorders^7^, schizophrenic disorders^8^, addictive behaviors^9,10^, obsessive-compulsive disorders^11^, attention deficit hyperactivity disorder (ADHD), and mood disorders^12^. Besides ID that is common, the heterogeneity in these potential ICD and DSM diagnoses indicates the limitations of these classifications, especially for comprehensive genetic models. The dimensional approach has, however, been supported by a rich literature for PWS that includes biological, neuroimaging, and behavioral characterizations of human disease combined with data from mice and cell models.

The diagnosis of PWS is currently made in the first months of life^13^ and the early follow-up of these patients has made it possible to describe the evolution in symptoms. This is especially the case for eating behavior, which generally shifts from anorexia in infancy to hyperphagia in childhood and adulthood following specific nutritional phases^14^. Moreover, therapeutic studies have observed the age-related effects—and therefore the neurodevelopmental steps (or phases)—on the therapeutic results, which have been well-documented in studies on the administration of intranasal oxytocin (OXT). The studies have revealed results that vary with the age at administration and have clearly identified an early biological window^15,16^.

Last, the clinical and pathophysiological research on PWS has paved the way for unraveling the functions of the candidate genes. Indeed, although the PWS chromosomal region encompasses several maternal imprinted genes, recent studies were able to connect some of them with specific symptoms and phenotypes. The lack of expression of the *MAGEL2* gene causes Schaaf–Yang disease, which includes social interaction deficits with autism spectrum disorders and early feeding deficits^17^. *SNORD116* is the minimal gene mutation associated with the PWS-like phenotype, including the shift from early anorexia to hyperphagia and social interaction impairments^18,19^.

Based on these observations, we hypothesized that the RDoC matrix would be a useful way to combine the data on the psychiatric manifestations of PWS and the genetic data on this disease, ultimately helping to identify new genetic markers to document the RDoC dimensions. To develop this point, we describe the current knowledge on PWS following the RDoC matrix structure.

## RDoC approach and PWS

### Positive valence

Positive valence systems include the response to positive motivational situations or contexts, such as reward seeking, consummatory behavior, and reward learning. They are composed of several constructs, such as reward responsiveness, reward learning, and reward valuation. PWS patients present an excessive response in the positive valence system for food.

#### Behavior

PWS patients display hyperphagia and food‐related behaviors that dramatically impair socialization and occupational performance, substantially deteriorate their quality of life and that of their relatives, and are responsible for significant morbidity and mortality especially linked to obesity development, complications, and gorging behavior^10^. Early diagnosis has led to better knowledge of the natural evolution of the disease and a description of the nutritional phases and eating behavior^14^. At birth, PWS newborns show poor appetite and sucking, with low weight gain and failure to thrive. Then, at about 2 years old, rapid and excessive weight gain precedes the manifestation of an excessive interest in food and a growing appetite, with ultimately the development of hyperphagia with poor satiety. The gradual rise in the focus on food and the continuing changes in appetite can be detected at ~4–5 years old, while independent food-seeking behaviors and visible hyperphagia becomes evident at 8–9 years. In the absence of proper care and management, early excessive weight gain followed by hyperphagia and impaired satiety results in severe obesity^10^. Moreover, the eating behavior presents specificities in reward control: that is, patients with PWS who are offered unlimited access to food will consume approximately three times more food and spend more time searching for and storing food than control subjects. The response to reward is also modified, as the preference for high-carbohydrate food is significantly higher in PWS patients than in obese patients. In addition, PWS patients are more likely to eat contaminated food and to make inappropriate food combinations than controls with and without intellectual disabilities.

#### Pathophysiology

The levels of ghrelin, and more generally the ghrelin system^20^, are abnormal in PWS. This gut peptide secreted by the stomach is the only orexigenic peptide. It is associated with eating behavior regulation, especially with the food craving, food hoarding and foraging, and food anticipatory activity that are displayed by patients with PWS, and it may be linked to the dopaminergic reward circuits. PWS patients display high circulating levels of ghrelin^21^ and this hyperghrelinemia starts early in life, before the occurrence of hyperphagia and obesity. Moreover, the circulating ghrelin levels in these patients remain elevated at all ages compared with age-matched controls and obese patients^21^. Modifications in the ratio of the two forms of ghrelin (acylated and unacylated) have been described in PWS. Acylated ghrelin (AG), considered the active form of the so-called “hunger hormone,” has orexigenic effects, and the unacylated (UAG) form has been reported to have the opposite effects. Infants with PWS have normal AG levels and high UAG levels, in contrast to later in life when they display obesity and hyperphagia. The relatively high UAG in infants may explain their poor appetite, which is comparable to anorexia^22^ and the high levels of AG with a relative deficit of UAG later on in life explain the hyperphagia and food craving. As a whole, total ghrelin levels are elevated in PWS at all ages.

Interestingly, the elevated total ghrelin levels with normal AG levels were also found in *Magel2*-knockout (KO) mice, which reproduce the postnatal anorexic phase observed in patients with PWS and show the same findings regarding circulating ghrelin^20^. In humans, *MAGEL2* mutations cause Schaaf–Yang syndrome, a PWS‐like syndrome characterized by an early feeding deficit and hypotonia, in association with severe autistic features, hormonal deficits and a lower occurrence of obesity compared to PWS.

Moreover, *Snord116* KO mice display an elevated ratio of proghrelin to mature ghrelin protein in stomach lysates. Of note, the antibodies used in conventional ghrelin assays detect both mature and unprocessed AG and UAG. However, the mice do not become obese, although display hyperphagia. Nevertheless, mediobasal hypothalamic deletion of *Snord116* induced in adult animals, which necessarily bypasses the neonatal period, resulted in both frank hyperphagia and, in a subset of animals, frank obesity^23^. These data are consistent with observations in cell models deleted for *SNORD116*, which display low levels of the hypothalamic gene *PSK1* encoding for the proconvertase PC1 enzyme, an endopeptidase responsible for the maturation of several hypothalamic and nonhypothalamic peptides, including ghrelin^24^. Thus, the reported hyperghrelinemia in patients with PWS and *Snord116* KO mice models likely reflects elevated circulating proghrelin and ghrelin.

#### Circuits

Modifications in brain activation were found to be related to eating behaviors in PWS, with significantly greater BOLD activation in the ventromedial prefrontal cortex in patients than controls when viewing food pictures^25^. In addition, these patients exhibit greater activation in the orbitofrontal cortex (OFC), medial prefrontal cortex (PFC), insula, hippocampus, and parahippocampal gyrus as they viewed the food pictures^26^. Compared with obese patients, PWS patients also demonstrate higher activity in reward/limbic regions (NAc, amygdala) and lower activity in the hypothalamus and hippocampus^27^. According to structural studies, PWS individuals show small gray matter volume in the OFC, a region that may be associated with both overeating and a propensity for detrimental food preferences, such as overindulgence in sweet foods. In addition, small gray matter volume is also noted in the caudate nucleus, a region that gives rise to disinhibition and a variety of affective disorders^28^.

### Negative valence

Negative valence systems are responsible for responses to aversive situations or contexts, such as fear, loss, or anxiety. They are composed of several constructs: acute threat (fear), potential threat (anxiety), sustained threat, loss, and frustrative nonreward. In PWS patients, negative valence has been associated with changes in routines or expectations.

#### Behavior

The aversive nature of change in PWS might be associated with frustrative nonreward and it may cause specific behavior patterns (e.g., temper outbursts)^29,30^. PWS patients frequently display anxiety and anxious moods. Indeed generalized anxiety disorder, social anxiety, and compulsive behaviors are more frequently diagnosed in PWS children than other children with intellectual disabilities^6,31^.

#### Pathophysiology

The genetic mutation of PWS encompasses the *SNORD* 115 gene responsible for changes in the edition and alternative splicing of the serotonin receptor 2 C (5-HT2cR)^32,33^. Moreover, one study reported abnormal serotonin turnover associated with PWS, with elevated concentrations of serotonin metabolites in the cerebrospinal fluid of children and adolescents^34^.

5-HT2cR is implicated in the regulation of a variety of physiological functions, such as mood, satiety, and reproduction, and aberrant 5-HT2cR signaling might be associated with anxiety, depression, and obesity. People with PWS present features, close to obsessive-compulsive behavior, that can be explained by dysregulation of the serotonergic system, and selective serotonin reuptake inhibitors are sometimes prescribed to ameliorate the symptoms^35,36^. Mice lacking expression of PWS locus genes exhibit alterations in specific 5-HT2cR-related behaviors along with increased RNA-editing of the 5ht2c pre-mRNA^37^. Moreover, mice with mutations of all the sites for edition of 5HT2c R display features comparable to PWS, including failure to thrive, decreased somatic growth, neonatal hypotonia, and reduced food consumption followed by postweaning hyperphagia^38^.

#### Circuits

PWS patients show small gray matter volume in the OFC that is linked to compulsive behavior^28^ and abnormal functional connectivity between the PFC and basal ganglia and within the subcortical structures that correlate with the presence and severity of obsessive-compulsive behaviors^39^.

### Cognitive systems

Cognitive systems are responsible for several cognitive processes. They are composed of attention (referring to processes that regulate access to capacity-limited systems), perception (referring to processes that perform computations on sensory data), declarative memory (referring to the acquisition or encoding, storage and consolidation, and retrieval of representations of facts and events), and language and cognitive control (referring to a system that modulates the operation of other cognitive and emotional systems and is engaged in the case of novel contexts). Intellectual disability is classically described in PWS as involving several cognitive systems, although the intellectual quotient (IQ) also depends on the type of mutation, and PWS subjects with maternal disomy have significantly higher verbal IQ scores than those with a deletion^40^.

#### Behavior

Attentional skill is weakened in the PWS population for both global and selective attention^41,42^. Immediate memory is also affected, with low scores^43^. However, when the task requires simultaneous visual processing, the scores are preserved^44,45^. PWS patients display learning difficulties especially in observational learning^46,47^. A deficit in executive function has been observed with impaired mental switching^30,48^, and impaired inhibition capacities have also been noted^49^. Concerning visual perception, some authors have argued that patient with PWS display higher skills to detect similarities which possibly explain their high performance in puzzle processing^45^. However, more recent study noted greater deficits verbal or visual concerning the sequential processes^42^.

#### Pathophysiology

Currently, the biological markers in the model of cognitive impairment and behavior suggests the effect of the genetic status on brain development, with the assumption that a genetic abnormality will influence neurodevelopment^30^. Nevertheless, the biological pathway that has been impaired during neurodevelopment has yet to be specified.

#### Circuits

During neurodevelopment, several brain regions show impairment in PWS: the frontal, parietal, and temporal lobes have lower local gyrification indices, and lower gyrification in the frontal lobes is correlated with lower IQ in children with PWS^50^.

### Social processes

The social processes mediate responses in interpersonal settings of various types and include the perception and interpretation of the actions and thoughts of others. These processes are composed of constructs of affiliation and attachment, social communication and perception, and understanding of self. PWS patients show deficits in emotion recognition, expression, and regulation and in social behaviors. The ability to establish appropriate social interactions entails several distinct processes. First, the social agent must recognize the others as “living persons,” then she/he has to recognize the other behaviors in terms of mental states and dispositions (i.e., “mentalizing” or “theory of mind”, finally she/he has to modulate the decision-making of her/his behavior. The outcome of these processes will likely lead the observer to adapt her/his own social behavior. The impairment in social interaction could impact one of several of these steps. Various tests have been developed to precisely identify the deficits in social skills such as Sally et Ann test and proposed scoring systems.

#### Behavior

PWS patients recognized joy in 90% of the cases presented to them but sadness in only 55%, anger in 40%, fear in 37%, surprise in 55%, and disgust in 43%^51^. These patients have an impaired ability to organize visual information into a coherent social story, have more difficulty making appropriate social attributions than do other patients matched for IQ^52^, and show impaired voice detection and face^53–55^. Moreover, PWS patients fare worse than Down’s syndrome patients in behavior with others and are less active in social organization^56^ in association with emotional lability^57,58^.

#### Pathophysiology

Event-related potential (ERP) research has shown that PWS patients demonstrate altered processing of, attention to and/or recognition of faces and their expressions^59^. In PWS, the pathophysiology underlying social withdrawal has been attributed to impaired and dysfunctional hypothalamic OXT neurons^57,60^, and studies have shown improvements in PWS patients after intranasal OXT administration^15,16^. *MAGEL2* KO mice, which also show impaired eating behavior at birth and social impairment close to autism, are likewise improved by postnatal intranasal OXT treatment. In the *MAGEL2* mice model, defects in OXT secretion, receptors and neurons have now been clearly established. In addition, the inactivation of *Magel2* suppresses OXT neuron activity through an altered synaptic input profile, with reduced excitatory and increased inhibitory currents^61^. Last, hypothalamic neurons derived from induced pluripotent cells (IPSCs) deleted for *SNORD116* display a dysfunction in OXT synthesis with a defect in OXT maturation^24^.

#### Circuits

PET scans of PWS patients have revealed a hypoperfusion in the anterior gyrus cinguli and the superior part of the temporal lobes, two regions implicated in theory of mind and empathy^62^. In addition, these patients show small gray‐matter volume in the OFC. In this brain region, two distinct populations of neurons were identified in mice, one related to feeding and the other to social response. This naturally feeding-responsive neuron population was causally linked to increased feeding behavior and the naturally social-responsive neuron population, which inhibits feeding, thereby indicating an association between social processes and eating behaviors^63^.

### Arousal/ regulatory systems

The arousal/regulatory systems are responsible for activating neural systems for various systems, as appropriate, and providing homeostatic regulation. They are composed of constructs of arousal, circadian rhythms, sleep, and wakefulness. PWS patients present sleep disorders and arousal dysregulation.

#### Behavior

PWS patients display sleep abnormalities with central sleep apnea predominating in infants and children^64^ and excessive daytime sleepiness with or without narcolepsy. Moreover, some patients continue to have difficulty with hypersomnia even after the sleep apnea has been treated, and the excessive sleepiness seems to be due to hypothalamic dysfunction^65,66^. In others, the sleep disorder is associated with a narcolepsy-like phenotype that includes sleep-onset REM periods and sometimes cataplexy^67^. PWS patients also show endocrine dysfunction with pituitary hormone deficits, which explains the growth retardation, hypogonadism, and metabolic and appetite dysregulation.

#### Pathophysiology

The sleep-wake cycle is regulated by two major processes: homeostatic sleep pressure, which is defined as the gradual accumulation of sleep factors (i.e., peptides, hormones, and neurotransmitters), and circadian rhythms, which can be linked to the feedback loop of the core time-keeping genes: Per, Cry, CLOCK, and BMAL1 and their target genes. These genes in turn mediate circadian timing in physiological processes, such as sleep, feeding, and energy balance^68^. In PWS, the lack of expression of *MAGEL2* and *SNORD116* has been noted to be associated with a shortened circadian rhythm and dysregulation of diurnally regulated gene expression, as well as with a further dysregulation of sleep and activity^69–71^. Moreover, orexin-A (hypocretin-1), a neuropeptide crucial for maintaining wakefulness, has shown an intermediate level in the cerebral spinal fluid of some PWS patients with excessive daytime sleepiness^72,73^.

Concerning homeostasis, mice deleted for *Snord116* display an increased energy expenditure, likely via the Neuropeptide Y (NPY) system in the hypothalamus^74^, the upregulation of AGRP, and the downregulation of Proopiomelanocortin (POMC)^19,75^ The lack of expression of *Magel2* in mice leads to dysregulation of leptin sensitivity in the POMC neurons, which thus fail to induce the fasting response^76^.

### Sensorimotor systems

The sensorimotor systems are primarily responsible for the control and execution of motor behaviors and their refinement during learning and development. They are composed of constructs for motor actions, agency and ownership, habits, and innate motor patterns. Sensory processing involves perceiving, organizing and interpreting information received through sensory systems (e.g., touch, smell, sight, auditory, taste, and vestibular) in order to produce an adaptive response. The term “sensory integration” refers to the ability to produce appropriate motor and behavioral responses to stimuli. PWS is characterized by motor and sensory impairment.

#### Behavior

PWS is characterized by motor system modifications with severe hypotonia in infancy. PWS patients in general have normal muscle size but low muscle mass and their grip strength is diminished by about 25%, and PWS children have lower maximal muscle power and force relative to their body weight^77,78^. The sensory system is also implicated with skin-picking, a pathological behavior particularly prevalent in PWS and previously found in 69–95% of young PWS patients^79^ and 81% of adults^80^. Hall et al. suggested that this skin-picking was maintained by automatically produced sensory consequences^81^, and Hustyi et al. proposed that it might serve as sensory stimulation^82^, with both thus suggesting an alteration in the sensory pathway. In addition, an impairment in multisensory integration has been described with a lower gain in sensitivity in a multimodal condition compared with a control condition in visual–auditive combinations^54^.

PWS patients also present an autonomic nervous system dysfunction with a lower resting diastolic blood pressure and significantly less change in diastolic blood pressure after standing, which correlates significantly with the body mass index (BMI)^83,84^. In addition, several cardiac autonomic parameters differ during sleep between PWS patients and gender- and age-matched controls, with the patients showing altered parasympathetic activity^84^. Moreover, thermoregulation problems, resulting in hypo‐ or hyperthermia, have been infrequently reported in children with PWS^85^. Last, patients with PWS present gastrointestinal autonomic disorders characterized by an increased prevalence of prolonged total gastrointestinal transit time^86^, and delayed gastric emptying has generally been reported^87^.

#### Circuits

Interoceptive dysfunction has been detected in PWS patients, who show higher pain thresholds than controls^88^ with increased functional connectivity across the anterior cingular/insula and frontal regions^89^ and abnormal GABA-A receptor binding within the insula^90^. Klabunde et al. noted that the functional activation of interoceptive circuits occurs during skin-picking, and the relationship between insula activation and the severity of skin-picking suggests that this behavior may be reinforced by interoceptive consequences^91^. In addition, treatment with N-acetylcysteine (NAC), a derivative of the amino acid cysteine, is thought to act by either modulating NMDA glutamate receptors or increasing glutathione, and it has been associated with improvement in skin-picking behaviors^92^.

PWS patients also show small gray matter volume in the supplementary motor area, the caudate nucleus and the cerebellum with a hyperperfusion of the cerebellum^62^. The small gray matter volume in the cerebellum, as well as in the motor and sensory cortices, may be related to the central hypotonia in PWS^28^. In addition, the supplementary motor area and the caudate nucleus contribute to the control of movement. Interestingly, OXT treatment not only improves feeding skills, but also enhances spontaneous movements and improves coordination skills^15^. Moreover, it has been demonstrated in the mice model that *Magel2* is required for normal strength and endurance and the maintenance of muscle mass^93^.

## Conclusion

This review summarizes the current knowledge on the psychiatric symptoms observed in PWS patients and offers a new perspective through the RDoC matrix (Table 1). This work suggests the interest of constructing a developmental trajectory by combining the data on PWS collected from physiological, brain imaging, mice, and cell model studies. The brain imaging studies in PWS are usually limited by their sample size but that some results are comforted by several animal models very helpful. The early diagnosis of PWS enables us to investigate the neurodevelopmental and environmental influences on symptom emergence, such as the first changes in appetite and weight, which appear at ~9 months in PWS, the age at which complementary foods are first introduced to an infant’s diet^94^. Early diagnosis also facilitates the study of how modulating the environment can influence symptom improvement. One example is the setting of an energy-restricted and well-balanced diet before PWS children begin to develop excessive weight gain, as this has been shown to be efficient to reduce weight gain and fat mass^95,96^. Importantly, the neurodevelopmental aspect is illustrated by the differences observed for early and late treatment with OXT that confirm the therapeutic window relative to brain development. This observation is relevant, with studies demonstrating OXT circuit development in animal models, and underlines the need to take into account the brain specificities over the time for treatment^97^.

**Table 1:** Summary of the RDoCMatrix filled in with current knowledge on PWS:AGRP, NAc, OXT, OFC, and POMC.

	Behavior	Pathophysiology	Brain neurocircuits	Specific gene in the 15q11–13 locus
Positive valence	Hyperphagia Food addiction	High blood level of ghrelin Impaired OXT system	Hyperactivation of ventromedial prefrontal region Hyperactivation of NAc and amygdala Hypoactivation in hypothalamus, hippocampus, and amygdala	*MAGEL 2 SNORD 116*
Negative valence	Tantrum Low frustrative tolerance Anxiety	High serotonin degradation Impaired editing and splicing of serotonin receptor 2 C	Hypoactivation of OFC Abnormal functional connectivity between the prefrontal cortex and basal ganglia	*SNORD 115*
Cognitive systems	Low attentional skills Executive functions deficits Deficits in multisensorial perception Low IQ	Neurodevelopmental hypothesis	Low gyrification index in the frontal lobe Low gyrification index in the parietal lobe Low gyrification index in the temporal lobe	?
Social processes	Deficits in emotion recognition Deficits in human voice and face recognition Difficulties in social interactions	Deficit in OXT production –OXT neuron degeneration –OXT processing impairment ­ altered synaptic input profile, with reduced excitatory and increased inhibitory currents in OXT neurons	Hypoperfusion in anterior cingulate region Hypoperfusion in the superior part of temporal lobe	*MAGEL 2 SNORD 116*
Arousal/regulatory systems	Central sleep apnea Hypersomnia Deficit in balance energy regulation Multiple pituitary hormone deficiencies	Impaired neuropeptide processing Upregulation of AGRP Downregulation of POMC Orexin-A dysregulation Leptin dysregulation	Hypoactivation in hypothalamus	*MAGEL* 2 *SNORD116* *Clock genes*
Sensorimotor system	Hypotonia Skin-picking Multimodal of sensory treatment impairment Autonomic nervous system dysfunction	Interoceptive circuit dysfunction	Increased connectivity between anterior cingulate region and frontal region Small volume of cerebellum Small volume of supplementary motor area Small volume of caudate nucleus Cerebellar hyperperfusion	?

The “?” symbol indicates that the knowledge is currently missing.

*PWS:AGRP* agouti-related protein, *NAc* nucleus accumbens, *OXT* oxytocin, *OFC* orbitofrontal cortex, *POMC* proopiomelanocortin.

One of the issues for research in psychiatry, especially translational research, is the constitution of homogenous groups for the same diagnosis. The advantage of approaches that use the data of rare genetic disorders is that they are able to identify specific relationships between an objective marker and clinical manifestation through comprehensive animal models. We suggest that these observations will help identify therapeutic targets and biomarkers in psychiatry as supported by the RDoC framework and its multidimensional exploration. This hypothesis is supported by the RDoC framework, which proposes the multidimensional exploration of psychiatric disorders.

Exciting perspectives can then emerge, as well as new therapeutic approaches including genetic or epigenetic therapy. Indeed, reactivation of the *SNORD116* gene in the *Snord116* KO mice model improves the PWS phenotype^98^. Obviously, many steps are required before envisaging these treatments; however, the current scientific advances have opened up this field (or this possibility)^99^.
